# 143. Use of First-Generation Cephalosporins in Patients with Serious Penicillin Allergies

**DOI:** 10.1093/ofid/ofab466.143

**Published:** 2021-12-04

**Authors:** Sydney E McNeill, Shauna Junco, Janessa Smith, Mallory C Cowart, Alejandro Jordan Villegas, Edgar Sanchez

**Affiliations:** 1 Orlando Health - Orlando Regional Medical Center, Orlando, FL; 2 Orlando Health, Orlando, Florida; 3 Orlando Health - Arnold Palmer Hospital for Children, Orlando, Florida

## Abstract

**Background:**

Penicillin allergies have a negative impact on patient outcomes due to utilization of second-line agents. Newer data suggests cephalosporins are well tolerated in penicillin allergies; however, none have solely evaluated anaphylactic penicillin allergies with first-generation cephalosporins. The purpose of this study was to evaluate the risk of any allergic reaction to first-generation cephalosporins compared to aztreonam in patients reporting anaphylaxis to an agent in the penicillin class.

**Methods:**

This was a retrospective cohort study with patients who reported “anaphylaxis” to a penicillin agent and received cefazolin, cephalexin, or aztreonam. The final analysis included 220 patients: aztreonam (n=81), cefazolin (n=81), and cephalexin (n=58) (Figure 1). IgE-mediated reactions (within six hours of antibiotic administration) were defined as any one of the following: anaphylaxis, angioedema, urticarial rash, hypotension, immediate airway compromise, or receipt of epinephrine, hydrocortisone, or diphenhydramine. Non-IgE mediated reactions (within thirty days of antibiotic administration) included delayed hypersensitivity reactions and other dermatologic reactions.

Figure 1: Patient Enrollment

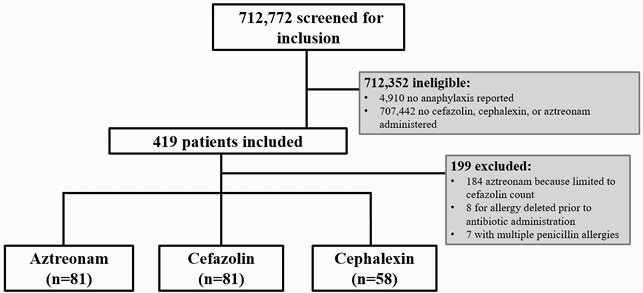

Patients admitted between January 1, 2013 to September 1, 2020 with a reported allergy of “anaphylaxis” to an agent in the penicillin class who received at least one dose of cefazolin, cephalexin, or aztreonam were screened for inclusion. Patients were excluded if the allergy was deleted from the electronic health record prior to antibiotic administration. All first-generation cephalosporin patients were included. Aztreonam patients were included in chronological order and limited to the number of included cefazolin patients.

**Results:**

There were less allergic reactions in the first-generation cephalosporin group compared to the aztreonam group, but this was not statistically significant (7% vs. 14%, *p*=0.077). There were fewer IgE-mediated reactions in the cephalosporin group (6% vs. 14%, *p*=0.046). No difference in allergic reactions was observed when comparing those who received a single antibiotic dose versus multiple doses within the cephalosporin and aztreonam groups, respectively (3% vs. 11%, *p*=0.082, 20% vs. 12%, *p*=0.451). Because cephalexin has a similar R1 side chain to aminopenicillins, five patients with an aminopenicillin allergy who received cephalexin were evaluated separately; none had an allergic reaction (Table 1, Table 2, Figure 2).

Table 1: Baseline Characteristics

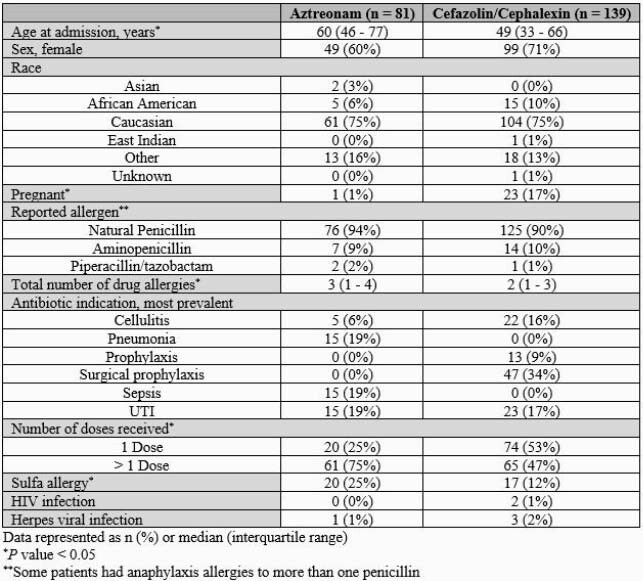

The median age was higher in the aztreonam group, and the majority of patients were female and Caucasian. There were significantly more pregnant females in the cephalosporin group, and the majority of patients reported a natural penicillin allergy.

Table 2: Outcomes

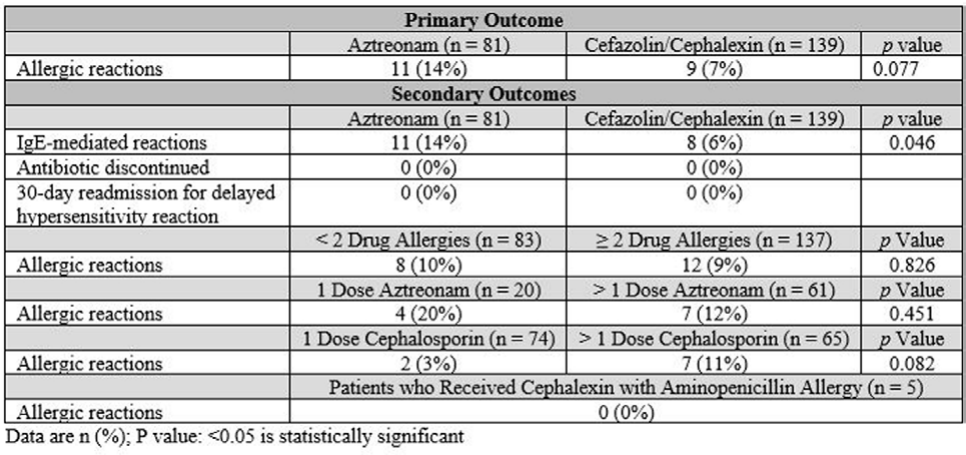

There were less allergic reactions (IgE or non-IgE mediated) in the first-generation cephalosporin group compared to the aztreonam group, but this was not statistically significant. Also, there were fewer IgE-mediated reactions in the cephalosporin group. There was no difference in allergic reactions in patients with two or more reported drug allergies compared to less than two drug allergies. No difference in allergic reactions was observed when comparing those who received a single antibiotic dose versus multiple doses within the cephalosporin and aztreonam groups. Of the five patients who received cephalexin and reported an aminopenicillin anaphylactic allergy, none had an allergic reaction. Additionally, there were not any patients readmitted within 30 days for delayed hypersensitivity reactions and no antibiotics were discontinued due to other documented adverse reactions.

Figure 2: Occurrence of Allergic Reactions

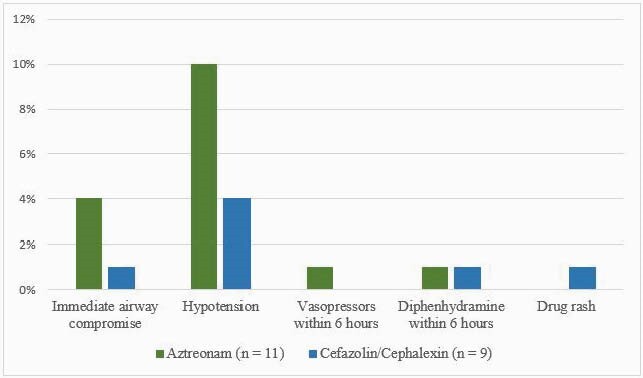

Of the patients who had allergic reactions in the cephalosporin and aztreonam groups, these included immediate airway compromise, hypotension with one patient in the aztreonam group receiving vasopressors within the pre-defined time frame, receipt of the non-standing rescue medication of diphenhydramine, and drug rash.

**Conclusion:**

There was no difference in the incidence of allergic reactions between the aztreonam and first-generation cephalosporin group, and fewer serious allergic reactions occurred in the cephalosporin group. This study suggests that cefazolin and cephalexin can safely be used in patients who report anaphylaxis to an agent in the penicillin class.

**Disclosures:**

**Janessa Smith, PharmD**, **Merck & Co.** (Employee)

